# Novel Compound Heterozygous *PKLR* Mutation Induced Pyruvate Kinase Deficiency With Persistent Pulmonary Hypertension in a Neonate: A Case Report

**DOI:** 10.3389/fcvm.2022.872172

**Published:** 2022-04-26

**Authors:** Sha Lin, Xintian Hua, Jinrong Li, Yifei Li

**Affiliations:** Key Laboratory of Birth Defects and Related Diseases of Women and Children of MOE, Department of Pediatrics, West China Second University Hospital, Sichuan University, Chengdu, China

**Keywords:** case report, genomic sequence, persistent pulmonary hypertension in the neonate, *PKLR*, pyruvate kinase deficiency

## Abstract

**Background:**

Pulmonary hypertension could be associated with pyruvate kinase deficiency (PKD). There are few reported cases of PPHN as the first clinical manifestation of PKD. Herein we report a rare case of PKD in which the patient exhibited persistent pulmonary hypertension in the neonate (PPHN), and genetic testing helped to rapidly identify an potential association.

**Case presentation:**

The patient was a newborn boy who suffered from severe dyspnea, extreme anemia, skin pallor, and hypoxemia. Repeated echocardiography indicated persistent severe pulmonary hypertension with a calculated pulmonary artery pressure of 75 mmHg, and right ventricular hypertrophy. The administration of nitric oxide significantly reduced the pulmonary artery pressure. Whole-exome sequencing revealed a compound heterozygous mutation consisting of c.707T > G and c.826_827insAGGAGCATGGGG. PolyPhen_2 and MutationTaster indicated that both the c.707T > G (probability 0.999) and c.826_827insAGGAGCATGGGG (probability 0.998) mutations were disease causing. PROVEAN protein batch analysis indicated that the associated p.L236R region was deleterious (score −4.71) and damaging (SIFT prediction 0.00), and this was also the case for p.G275_V276insEEHG (deleterious score −12.00, SIFT prediction 0.00). Substantial structural changes in the transport domain of the protein were predicted using SWISS-MODEL, and indicated that both mutations led to an unstable protein structure. Thus, a novel compound heterozygous mutation of PKLR-induced PKD with PPHN was diagnosed.

**Conclusion:**

The current study suggests that molecular genetic screening is useful for identifying PPHN, particularly in children with metabolic disorders. In patients exhibiting unexplained hyperbilirubinemia combined with severe pulmonary hypertension, PKD might be a potential possible alternative explanation. Genetic screening is helpful for identifying genetic causes of pulmonary hypertension, especially in patients with PPHN. This report expands the mutation spectrum of the *PKLR* gene, and contributes to the genotype-phenotype map of PKD.

## Introduction

Persistent pulmonary hypertension in the neonate (PPHN) is a severe condition. Generally, life – threatening infection, developmental disorders, and cardiac malformation are considered the main causes of PPHN. Notably, however, metabolic disorders are increasingly being identified as involved in the development of PPHN. Genetic abnormalities have been reported in association with refractory PPHN, such as mutations in surfactant protein B and the ATP-binding cassette protein A3. Such mutations may lead to insufficient surfactant function, and induce PPHN. Beyond pulmonary disease, several inherited systemic hematological and metabolic disorders are reportedly associated with pulmonary hypertension, including primary thrombocytosis, polycythemia vera, Gaucher disease, glycogen storage disease, and pyruvate kinase deficiency (PKD). Notably, however, few patients with hematological diseases have been identified with pulmonary hypertension in the neonatal period. Due to hypoxia and hemolysis, PPHN may present as a severe complication with hyperbilirubinemia.

Pyruvate kinase deficiency is the most common defect affected by the glycolytic pathway, and it can result in severe congenital hemolytic anemia (1). With the development of genetic sequencing, mutations in the pyruvate kinase L/R (*PKLR*) gene located on chromosome 1q21 have been identified as genetic causes of PKD (2, 3). To date > 300 mutations have been reported. Differences in residual red blood cell (RBC) enzyme between homozygotes (>25%) and heterozygotes (40–60%) of *PKLR* mutations have also been described (1). PKD has been regarded as a heterogeneous disease caused by reduced PK activity and PKLR variants (4–6). PKLR participates in glycolysis as a catalyst involved in the transphosphorylation of phosphoenolpyruvate into pyruvate and ATP, which is an irreversible component of glycolysis. Previous reports suggest that the generation of ATP in RBCs contributes to maintaining their structural and functional integrity throughout their lifecycle, and deficiency of pyruvate kinase activity could result in failure of ATP production. Potential consequences include membrane plasticity loss, cellular dehydration, and the premature destruction of RBCs in the spleen or liver, leading to severe refractory hyperbilirubinemia and anemia in neonates.

In most reported cases of PKD the crucial clinical symptoms were severe anemic phenomena and substantial elevation of indirect bilirubin (IDIL) (5). Pulmonary hypertension is considered a rare and life-threatening complication of PKD, while PPHN has been seldom reported. Recent reports indicate that the incidence of pulmonary hypertension in PKD patients is relatively low, and that it is predominantly observed in adolescents (>3%) and adults (>5%). Hitherto there have been no reported cases in newborns, but a statement provided limited evidence on pulmonary hypertension during neonatal period (5). Herein we describe a case of severe hyperbilirubinemia and PPHN diagnosed soon after birth. Metabolic screening and genetic testing helped to identify a novel heterozygous mutation of *PKLD*, guiding further optimal therapeutic strategies. This is the first report of PKD with PPHN, and expands the known mutations associated with PKLD, and it also suggests that rapid genetic testing to distinguish between potential causes of PPHN in complex situations would be beneficial.

## Case Presentation

### Ethics Compliance

The study was approved by the ethics committee of the West China Second Hospital of Sichuan University (approval number 2014-034). Written informed consent was obtained from the patient’s parents prior to performing WES, and for the inclusion of the patient’s clinical and imaging details in publications.

### History of Illness and Physical Examination

The patient was a newborn boy who was admitted to our hospital 5 h after birth suffering severe dyspnea, extreme anemia, skin pallor, and hypoxemia. He was born at 37 + 6 gestational weeks, and his birth weight was 2.4 kg. Due to substantial intrauterine distress, meconium-stained amniotic fluid, and torsion of umbilical cord a cesarean had been performed to prevent further injuries. The respective Apgar scores were 7, 8, and 9 at the 1st, 5th, and 10th minutes after birth. Physical examination revealed mild jaundice, weak breath sounds in bilateral lungs, and dull cardiac sound. An enlarged liver was evident 5.0 cm under the lower rib and 3.5 cm below the xiphoid process, with a blunt edge, and the spleen was 3.0 cm under the rib. He had hypomyotonia of limbs, decreased primary reflex, and cold extremities with a capillary refill time of 3 s.

### Laboratory and Radiological Results

**FIGURE 1:**
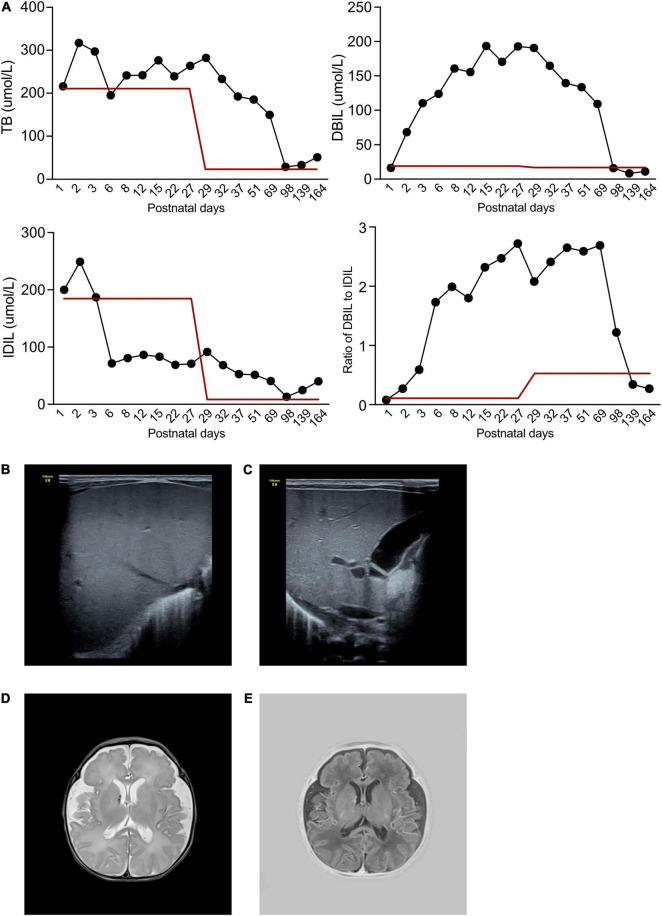
Clinical and radiology manifestation in the current proband. **(A)** The patient exhibited abnormal bilirubin in plasma, while the total bilirubin remained high during his illness. At the beginning, DBIL elevated rapidly to a high level, while IDIL remained normal. However, after 3 months follow-up, DBIL dropped to normal level, and IDIL increased. **(B)** Hepatic ultrasound demonstrated a normal density of liver. **(C)** Gallbladder demonstrated a normal morphology. **(D,E)** The cerebral MRI identified hematoma near right cerebral ventricle.

The patient demonstrated severe anemia within a short time postnatally (hemoglobin 56 g/L, n.v. > 170 g/L), and reduced platelets (67 × 10^9^/L, n.v. > 292 × 10^9^/L). White blood cells were elevated (92.6 × 10^9^/L, n.v 9.0–30.0 × 10^9^/L), as was C-reactive protein (12.9 mg/L, n.v. < 8.0 mg/L). Natriuretic peptide was 4379.8 pg/mL, indicating heart failure. Biochemistry tests demonstrated an elevation of total bilirubin (216.4 μmol/L, n.v. < 210.0 μmol/L), IDIL of 200.1 μmol/L (n.v < 193.0 μmol/L), and direct bilirubin (DBIL) of 16.3 μmol/L (n.v. < 10.0 μmol/L). IDIL dropped to approximately 50–80 μmol/L from the 3rd postnatal day (Figure 1A), but DBIL had increased to 193.4 μmol/L by the 15th postnatal day (Figure 1A). In tests for specific pathogens, quantitative polymerase chain reaction (qPCR) to detect pathogenic bacteria, viruses, and fungi, TORCH IgM antibody screening, group B *Streptococcus* testing were negative. Besides, the aerobic or anaerobic culture cultures of blood, septum, urine and cerebral spinal fluid were all negative. So that, non-specific pathogen had been identified.

Repeated echocardiography indicated persistent severe pulmonary hypertension with a highest calculated pulmonary artery pressure of 75 mmHg, with right ventricular hypertrophy at the 3rd day after birth. X-rays depicted patchy shadows in the lungs. Hepatic ultrasound indicated normal liver density (Figure 1B) and normal gallbladder morphology (Figure 1C), thus liver fibrosis and biliary atresia were ruled out as causes of the elevated DBIL. Cerebral magnetic resonance imaging revealed hematoma signs in the right cerebral ventricle (T2 image in Figure 1D and T1 image in Figure 1E).

Given the above laboratory and radiological results in conjunction with his clinical manifestations, the dominant concerns were PPHN and unexplained continuously elevated DBIL. Thus, metabolic disorder was highly suspected. Comprehensive metabolic function profiling analyzing organic acids in urine revealed large proportions of α-keto-b-methylvaleric acid, methylglutaric acid, hydroxyphenylacetic acid, fumarate, acetoacetic acid, and ketoisocarproate, which were strongly indicative of a metabolic disorder.

### Molecular Results

**FIGURE 2:**
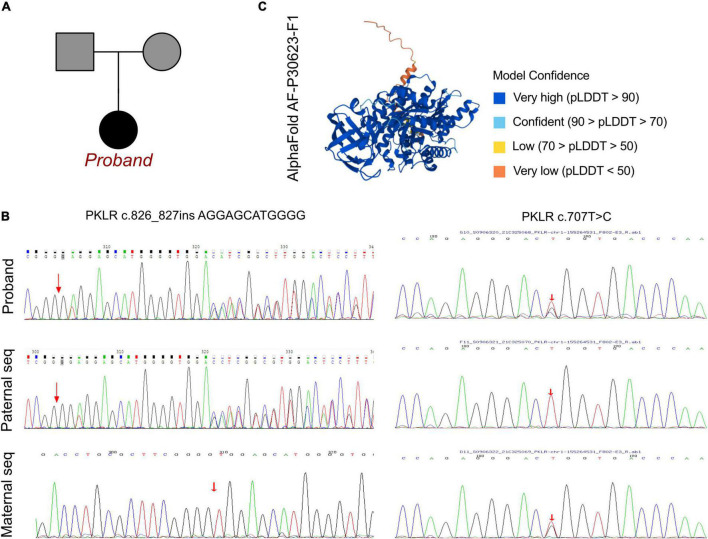
The *PKLR* mutations in this family. **(A)** Family pedigree in the current proband. **(B)** Sanger sequencing validation of the current proband and his parents, revealing the maternal carrier of *PKLR* c.707T > C (p.L236R) and his paternal carrier of *PKLR* c.826_827insAGGAGCATGGG. The current proband exhibited PPHN and metabolic disorder in neonatal period and congenital non-spherocytic hemolytic anemia with a novel compound heterozygous mutation of *PKLR*. **(C)** The crystal structure of full length of AA sequence built by AlphaFold 2.0.

A peripheral blood sample was obtained from the patient in an ethylenediaminetetraacetic acid anticoagulant blood sample tube then stored at 4°C for less than 6 h. DNA was extracted using the Blood Genome Column Medium Extraction Kit (Tiangen Biotech, Beijing, China) in accordance with the manufacturer’s instructions. Protein-coding exome enrichment was performed using the xGen Exome Research Panel v1.0. WES was performed using the NovaSeq 6000 platform (Illumina, San Diego, CA, United States). The patient’s parents reported that there was no known family history of pulmonary hypertension or PKD, and no other relatives in his family exhibited any potential symptoms (Figure 2A). PPHN was the primary consideration in this patient, as well as substantial elevations in total bilirubin, DBIL, and IDIL, consistent with metabolic disorder. Therefore, after ruling out other possible causes a genetic metabolic disorder was strongly suspected. WES was performed using the Illumina NovaSeq 6000 platform, followed by analysis of the variable genes involved in metabolic diseases, and a compound heterozygous mutation of c.707T > G and c.826_827insAGGAGCATGGGG were identified, both located in exon 6 of the *PKLR* gene. These data were confirmed by Sanger sequencing, and the patient’s mother carried the heterozygous variant of c.707T > G, and his father carried the c.826_827insAGGAGCATGGGG frame insertion as a heterozygous variant (Figure 2B).

To elucidate the molecular architecture of the human *PKLR* gene, MutationTaster with R software was used to predict the pathogenicity of *PKLR* c.707T > C and c.826_827insAGGAGCATGGGG. The effects of these mutations on protein structure were also assessed via PROVEAN protein batch with Provean score and SIFT prediction. A crystal structure was built based on amino acids 47–574, and formatting of the complex of pyruvate kinase isoform L-type with phosphorylated Ser113 (pS113). The AlphaFold protein structure database^1^ tool was used to predict a protein crystal structure containing amino acids 1–574. The protein structure of PKLR has been built and named AF-P30623-F1 (7, 8). Modeling analysis was performed using the online software SWISS-MODEL^2^ to visualize and analyze the altered amino acid sequence and stability of PKLR with the 6eck.1.A template. The capability of the protein structure was estimated using Ramachandran Plots. The signature vector that was ultimately generated was used to train the predictive classification and regression model for calculating the change in Gibbs folding free energy (ΔΔG) induced by the mutations shown in the ensemble variance figures.

**FIGURE 3:**
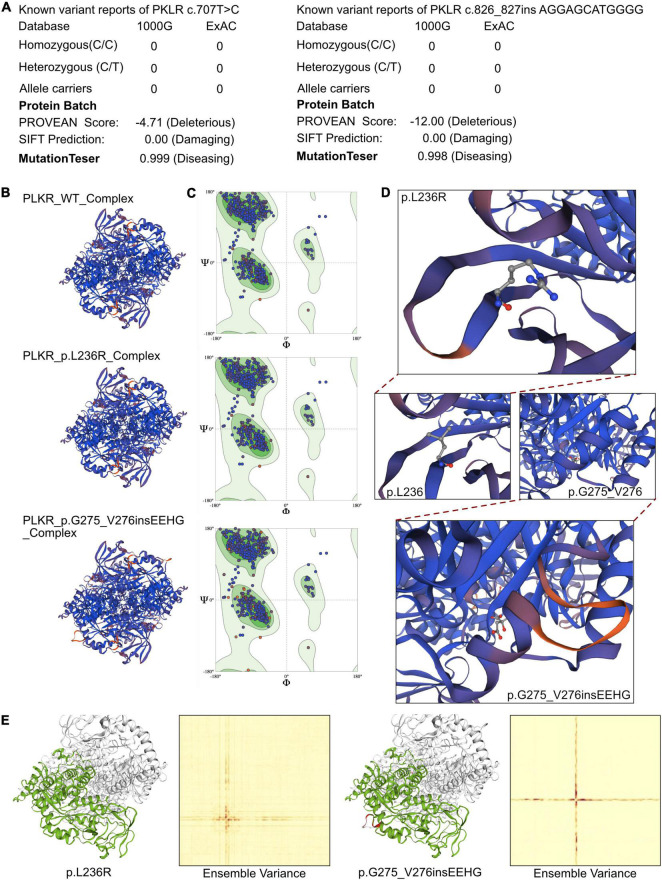
The effects of *PKLR* c.707T > C and c.826_827insAGGAGCATGGG mutations on the molecular structure of the protein. **(A)** The reported cases of the identified mutations in database 1000G and ExAC. And the PROVEAN protein batch and MutationTaster scores revealed the two newly identified mutations would be protein damaging and diseasing causing variants. **(B)** Individual crystal structures of wild type, p.L236R and p.G275_V276insEEHG according to the 36eck.1.A model template. **(C)** Ramachandran plots of AA with wild type, p.L236R and p.G275_V276insEEHG. **(D)** The detailed space structure between wild type and mutant proteins. **(E)** The comparisons of free energy on crystal structure of wild type sequence and p.L236R and p.G275_V276insEEHG variants, respectively.

The crystal structure of the full amino acid sequence is shown in Figure 2C. According to updated data in ExAC and 1000G neither of the mutations had been reported, indicating that they were novel variants (Figure 3A). Verification via Rare Exome Variant Ensemble Learner, SIFT, PolyPhen_2, and MutationTaster indicated that both the c.707T > G (probability 0.999) and c.826_827insAGGAGCATGGGG (probability 0.998) mutations were disease causing (Figure 3A). PROVEAN protein batch indicated that the p.L236R protein was deleterious (Provean score −4.71) and damaging (SIFT prediction 0.00), as was the p.G275_V276insEEHG protein (deleterious Provean score −12.00, damaging SIFT prediction 0.00) (Figure 3A). According to the American College of Medical Genetics the genetic variant of c.826_827 ins AGGAGCATGGGG was a frameshift with uncertain pathogenicity (PM2 + PM4), and the c.707T > G mutation was a missense mutation with uncertain pathogenicity (PM2 + PP3), and both were related to PKD.

SWISS-Model was used to assess the crystal structure of PKLR due to the identified mutations. The altered crystal structures of PLKR complexes are shown in Figure 3B. Generally there were no significant changes in crystal structure in the PKLR p.L236R complex, and an additional loop was identified in the PKLR p.G275_V276insEEHG complex (Figure 3B). Notably, however, differences in Ramachandran plots were observed in both PKLR p.L236R and PKLR p.G275_V276insEEHG compared to wild-type (Figure 3C). Detailed structures including changes in positions in p.L276R and p.G275_V276insEEHG are shown in Figure 3D. Structural comparisons were completed for both mutations, and are shown in Figure 3E. Ensemble variance revealed free energy changes due to the two mutations. The above analyses indicated that both of the mutations identified could alter the transcription of the *PKLR* gene, and damage the functional protein structures of the PKLR complex. Thus, a compound heterozygous mutation that was the likely cause of PKD was identified in the patient.

### Treatment and Clinical Outcome

During the neonatal period the patient was administered antibiotic therapy including ceftazidime and ampicillin. Mechanical ventilation, recurrent red blood cell and plasma transfusions, catecholamine, and nutritional support were administrated to save his life. After a series of positive treatments had been administrated to correct his severe anemia, alleviate hypoxia stress, and control infection, echocardiography still recorded pulmonary hypertension with a relative reduced pressure around 55–60 mmHg, which was different from other PPHNs induced by intrauterine stress and significant infection. So that, we considered the development of PPHN might be related with PKD, and nitric oxide inhalation had been provided to relieve PPHN. After the nitric oxide therapy, the pulmonary artery pressure dropped to 30–35 mmHg at the end of neonatal period. Furthermore, the pulmonary artery pressure decreased to normal of 20–25 mmHg at the age of 6 months postnatally.

IDIL increased again, however, whereas DBIL dropped to a normal level during 3 months of follow-up. The patient required RBC transfusions once a month, consistent with the classic clinical manifestation of PKD. The present case included PKD onset with PPHN and severe metabolic disorder, intrauterine distress, and perinatal extreme hypoxia. The typical clinical symptoms of congenital non-spherocytic hemolytic anemia appeared during follow-up. This case indicates that perinatal exposure to adverse environments may induce the onset of PKD with genetic deficiency of *PKLR*, resulting in various manifestations. Thus, early metabolic screening and WES may help to distinguish the disease based on molecular diagnosis, which could distinguish between truly positive and false positive PKD.

At present, the patient has been carefully followed by almost 18 months without any significant complications. And there was no obviously developmental retardation, cognitive function and motor movement delay. He received blood transfusion every 1.5 month, and 2 times blood test had been provided. To monitor the status of his cardiovascular function and pulmonary artery pressure, echocardiography had been involved every 2 months. After fully informed the patient’s parents, the patient would receive allogeneic hematopoietic stem cell transplantation around 3–5 years age.

## Discussion

The *PKLR* gene encodes RBC isoenzymes, and associated clinical symptoms are usually limited in RBCs. *PKLR* mutation leads to PKD. Hepatic enzyme activity is protected in hepatocytes, or compensated by the PK-M2 isoenzyme encoded by the *PKM* gene, which provides an explanation for degrees of diversity of anemia. Zanella et al. (9) reported that the clinical presentation is not entirely dependent on molecular variants, but reflects a combination of genetic background, concomitant functional polymorphisms of other enzymes, and ineffective erythropoiesis. (3) reported that *PKLR* variants did not contribute to intrauterine lesions. Surgical effects, infection, and hypoxia attacks may enhance the severity of PKD (10). PKD could be misdiagnosed due to its various clinical manifestations. In some patients the phenotype is nearly normal, whereas others exhibit extreme anemia and hyperbilirubinemia. Cardiopulmonary dysfunction is a life - threatening complication associated with PKD. PKD management guidelines usually recommend cardiovascular screening for outcome evaluation. In general, pulmonary hypertension is observed in 3% of adolescent PKD patients, but the incidence increases to 5% in adults (11). Several reviews indicate that pulmonary hypertension occurs in the neonatal phase, but none of them provide a convincing incidence rate. The average age that PKD-related pulmonary hypertension is diagnosed is 39.2 years (11). Thus, PPHN is seldom considered as the first clinical manifestation of PKD. In the current patient it was initially difficult to distinguish PKD from PPHN. Timely metabolic screening facilitated identification of the disorder, however, and prompted genetic testing, which resulted in a diagnosis of PKD at very early stage postnatally. PKD and other hemolytic anemia would lead to pulmonary hypertension in long term due to nitric oxide deficiency, oxidative stress, and hypercoagulability (12). And, the PPHN could be mainly induced by anemia, infection, intrauterine stress and meconium stained liquor. To this patients, we detected PPHN by routine echocardiography after birth and the highest pulmonary artery pressure was 75 mmHg. However, pulmonary pressure only reduced to 55–60 mmHg after anemia and infection had been controlled. Thus, nitric oxide inhalation had been provided, and the pulmonary artery pressure went down to 30–35 mmHg. Finally, the pulmonary artery pressure decreased to normal at 6 months old during follow-up. According to previous statement from Rachael F. Grace and Wilma Barcellini (5) indicated that PKD could induce critical anemia intrauterine, which would lead to evidence of neonatal pulmonary hypertension or stroke. So that we attempt to associated the presentation of PPHN with PKD based on the following reasons: (1) the patient suffered significantly severe anemia prenatally; (2) the pulmonary hypertension still remained after the anemia, infection and other conditions controlled; (3) the administration of nitric oxide significantly reduced the pulmonary hypertension; and (4) the pulmonary hypertension remained beyond neonatal period.

With respect to PKD’s etiology, perinatal hypoxia and infection could result in insufficient intracellular ATP supplementation. Elevation of arachidonic acid cyclase enhances the production of prostaglandins such as TXA2 and PGF2α, and increased lipoxygenase promotes the formation of leukotriene (13). Production of endothelium-derived contraction factor increases, in conjunction with a reduction in endothelium-derived relaxing factor, promoting pulmonary vasoconstriction. Histamine, angiotensin, and the consumption of nitric oxide caused by hemolysis are also involved in the generation of PPHN in neonatal PKD with hypoxia. Proliferation of vascular smooth muscle would contribute to PPHN.

Hyperbilirubinemia reportedly has some protective effects in patients with physiological jaundice and early-onset neonatal sepsis (14), but the sustained reversal of IDIL and DBIL elevation is also thought-provoking. Identifying the causes of persistent hyperbilirubinemia is critical in such patients. In the present patient substantially increased DBIL in the early phase was considered a consequence of PKD. Elevated IDIL was the major contributor to hyperbilirubinemia, however, which is the opposite of the classic clinical PKD manifestation. Based on genetic testing and molecular diagnosis, PKD was considered a confirmed diagnosis. An optimal therapeutic strategy was continuously provided to the patient. Integrated treatment to reduce IDIL was administered after excluding biliary atresia. During follow-up the IDIL level dropped to normal, and continuous hemolysis became the dominant concern, and also indicated the accuracy of the diagnosis of PKD. Severe perinatal hemolysis and hypoxia can evidently induce hepatic injury and damage intralobular canaliculi, which can result in elevated IDIL (15).

Neonatal jaundice and hyperbilirubinemia are commonly seen in infants with PKD, and can rapidly progress to hepatocellular damage and synthetic dysfunction. Early diagnosis is therefore crucial, but it is not easy to achieve. In the current patient WES was performed to facilitate an early molecular diagnosis. With the development of genetic sequencing, more than 300 *PKLR* gene mutations located on chromosome 1q21 have been confirmed to induce PKD. Thus, PKD could be definitively diagnosed even in neonates only exhibiting partial PKD-related symptoms. Compared to traditional analysis, WES helps to identify the radical fact of common symptom, such as hyperbilirubinemia and pulmonary hypertension. Molecular diagnosis contributes to identifying rare diseases, and providing targeted therapeutic strategies. To date the present patient has been given regular transfusions, and has not suffered iron overload, heart failure due to extreme anemia, or other complications, demonstrating the advantages of early genetic testing to identify rare diseases.

## Conclusion

In conclusion, there are few reported cases of PKD presenting with PPHN. In patients exhibiting unexplained hyperbilirubinemia combined with severe pulmonary hypertension, WES should be considered as a possible approach to reach a molecular diagnosis. Molecular genetic screening is helpful for identifying the genetic causes of pulmonary hypertension with metabolic disorders. The current report expands the mutation spectrum of the *PKLR* gene, and contributes to the genotype-phenotype map of PKD.

## Data Availability Statement

The original contributions presented in the study are included in the article/supplementary material, further inquiries can be directed to the corresponding authors.

## Ethics Statement

The studies involving human participants were reviewed and approved by the Ethics Committee of West China Second Hospital of Sichuan University (2014-034). Written informed consent to participate in this study was provided by the participants’ legal guardian/next of kin. Written informed consent was obtained from the individual(s), and minor(s)’ legal guardian/next of kin, for the publication of any potentially identifiable images or data included in this article.

## Author Contributions

YL and JL were the patient’s physicians. SL and XH reviewed the literature and contributed to manuscript drafting. YL conceptualized and designed the study, coordinated and supervised data collection, and critically reviewed the manuscript for important intellectual content. SL, YL, and JL were responsible for the revision of the manuscript for important intellectual content. All authors issued final approval for the version to be submitted.

## Conflict of Interest

The authors declare that the research was conducted in the absence of any commercial or financial relationships that could be construed as a potential conflict of interest.

## Publisher’s Note

All claims expressed in this article are solely those of the authors and do not necessarily represent those of their affiliated organizations, or those of the publisher, the editors and the reviewers. Any product that may be evaluated in this article, or claim that may be made by its manufacturer, is not guaranteed or endorsed by the publisher.

## Footnotes

1
https://alphafold.ebi.ac.uk/

2
https://swissmodel.expasy.org/
